# Natural Products as a Pipeline for Next-Generation Neurodegenerative Drugs: From Single-Target Failure to Multi-Target Opportunity in Alzheimer’s and Parkinson’s Disease

**DOI:** 10.3390/molecules31091489

**Published:** 2026-04-29

**Authors:** Solomon Habtemariam

**Affiliations:** Pharmacognosy Research & Herbal Analysis Services UK, 124 City Road, London EC1V 2NX, UK; s.habtemariam@herbalanalysis.co.uk

**Keywords:** neurodegenerative diseases, Alzheimer’s disease, Parkinson’s disease, drug discovery, one drug–one target, polypharmacology, multi-target drug design, network pharmacology, translational strategies

## Abstract

Neurodegenerative diseases such as Alzheimer’s disease (AD) and Parkinson’s disease (PD) represent some of the most complex and therapeutically challenging disorders in modern medicine. Despite decades of research, the traditional one drug–one target paradigm has largely failed to deliver disease-modifying therapies. Increasing evidence suggests that these complex diseases arise from interconnected pathological networks involving protein aggregation, oxidative stress, mitochondrial dysfunction, neuroinflammation, and synaptic loss. In this context, natural products (NPs) have re-emerged as a promising pipeline for next-generation therapeutics. Unlike conventional small molecules, NPs inherently exhibit polypharmacology, targeting multiple pathways simultaneously. Recent advances (2019–2026) demonstrate a paradigm shift, from crude NPs and single-mechanism compounds toward engineered derivatives, network pharmacology, and multi-target drug design. Using AD and PD as case studies, this review critically evaluates how NPs are redefining drug discovery by highlighting key emerging NPs, translational strategies, and future directions.

## 1. Introduction

Neurodegenerative diseases (NDs) are characterised by progressive loss of neuronal structure, function and neuronal cell death through pathological cascades collectively known as neurodegeneration. With an estimated global prevalence of 57 million in 2021, the best example of an ND is Alzheimer’s disease (AD), which contributes to 60–70% of dementia cases [1]. According to the WHO, AD is the seventh leading cause of death globally and one of the major causes of disability and dependency among older people. The second common form of NDs is Parkinson’s disease (PD), which is associated with motor symptoms (slow movement, tremor, rigidity, and walking imbalance) along with other complications such as cognitive impairment and mental health disorders, as well as pain and other sensory disturbances. The prevalence of both diseases has drastically increased over the last decades; for example, PD cases have doubled in the past 25 years [2] while dementia cases are doubling every ~20 years [3]. Despite extensive investment in drug discovery research in the field, therapeutic success against AD and PD has been limited, with most approved drugs only providing symptomatic relief. A handful of pharmacologic agents used for AD include cholinesterase inhibitors (e.g., donepezil, rivastigmine and galantamine), NMDA (*N*-methyl-D-aspartate) receptor antagonists (e.g., memantine) and the now-emerging disease-modifying immunotherapies such as lecanemab and donanemab. In the latter case, the approval of antibodies targeting amyloid plaques as therapeutic agents gives further hope that the amyloid-based therapeutic approach still holds promise for the treatment of AD. The gold standard pharmacological therapeutic approach for PD is based on levodopa (with carbidopa) which underpins the therapeutic principle of boosting dopamine synthesis preferentially in the brain. Other drugs, including monoamine oxidase-B (MAO-B) inhibitors (e.g., selegiline, rasagiline, and safinamide) to prevent the breakdown of dopamine, or its precursor levodopa by using catechol-*O*-methyltransferase (COMT) inhibitors (e.g., entacapone, tolcapone, and opicapone), are also available in PD pharmacotherapy. It remains the case, however, that there is no drug of cure for either AD or PD. Unlike many other diseases (e.g., cancer), the high rate of clinical failure for these NDs (particularly AD) underpins our incomplete understanding of their pathophysiology, as well as the reliability of biomarkers and animal models used in our studies.

A central reason for the failure of many clinical trials in NDs lies in our reliance on the traditional reductionist drug discovery paradigm, which assumes that modulating a single molecular target can halt disease progression. However, AD and PD are now understood as multifactorial network disorders, involving overlapping and interdependent pathological mechanisms. With long history of usage in traditional medicine, natural products (NPs) offer a fundamentally different approach in NDs therapy. Their chemical diversity and evolutionary optimisation enable them to simultaneously interact with multiple biological targets. Recent research further highlights their potential, not merely as general antioxidants or nutritional supplements, but as multi-target disease-modifying agents. This review assesses the common pitfalls of historical drug discovery approaches and the reemerging potential of NPs as a pipeline for next-generation drugs in NDs therapy.

## 2. Failure of the Single-Target Paradigm in AD and PD

The cholinergic hypothesis of AD established since the 1970s was underpinned by pioneering research showing the selective loss of central cholinergic neurons in AD patients. The study by Davies and Maloney [4] showing significant reduction in the activity of choline acetyltransferase (ChAT) in the brains of AD patients and subsequent studies [5]) demonstrating the association of AD and senile dementia with loss of neurons in the cholinergic basal forebrain served as the foundation for linking the loss of cholinergic activity to cognitive decline. Hence, the cholinergic hypothesis of AD is based on the principle that acetylcholine (ACh) is a primary neurotransmitter in memory and cognition, and its decreased activity in AD may be compensated by prolonging the activity of ACh using drugs such acetylcholinesterase (AChE) inhibitors. Accordingly, drugs like donepezil, rivastigmine, and galantamine can provide some symptomatic relief and indeed improve cognitive function and manage behavioural symptoms. They do not, however, halt the disease progression. As ACh deficiency is critically associated with AD, dopamine is a critical neurotransmitter in PD as progressive deletion of dopamine-producing neurons in the substantia nigra causes its characteristic symptoms. Based on findings from the early 1960s [6,7], dopamine replacement therapy, primarily using levodopa, is highly effective in providing symptomatic relief for the motor features of PD. Unfortunately, such therapies do not stop, slow, or reverse the underlying disease progression in PD. A combination therapy with carbidopa ensures the inhibition of peripheral metabolism of levodopa, thereby allowing levodopa to reach the brain while decreasing side effects like nausea and vomiting. Monoamine oxidase-B (MAO-B) inhibitors (e.g., selegiline, rasagiline, and safinamide) in PD therapy enhance the dopamine levels by blocking its breakdown while increasing the efficacy of levodopa therapy by inhibiting COMT enzyme (e.g., by entacapone), also providing therapeutic benefit.

All these drugs designed through one drug–one target approach improve NDs symptoms but have failed to halt the disease progression and/or progressive neuronal losses. As evidenced by the phenserine research by Axonyx in 2005 (see Section 6.1), many drugs of this kind that showed promise in in vitro, in vivo, and even up to phase II clinical trial failed in late-stage (phase III) trials due to lack of efficacy. The explanation lies in the inherent problems of a single-target approach, which is subject to the following limitations:

- The one-drug–one-target often fails to produce long-lasting efficacy in complex diseases, such as NDs, because it does not account for multiple interconnected pathways within normal biological and pathophysiological conditions. When one target is inhibited, parallel, redundant, or compensatory pathways often take over, preserving the disease state. Hence, a single-target approach does not address the biological redundancy in complex diseases.
- Given the above said complexity of NDs, targeting one node (molecule, protein, or cell type) does not disrupt the entire pathological cascade. Neurodegeneration involves interacting mechanisms such as pathological marker protein aggregation leading to oxidative stress and neuroinflammation, and this intern further aggravates protein aggregation. Targeting only aggregation ignores the oxidative/inflammatory feedback loop. The cascading network theory of AD spectrum [8] assumes that neurodegeneration may begin in specific hubs (like the default mode network in AD) and may shift the burden to other systems. Misfolded proteins (like amyloid beta (Aβ), tau, and α-synuclein) may spread across neuronal networks in a prion-like manner, a mechanism now thought to be involved both in AD and PD [9,10]. Hence, inhibiting one part of this process (e.g., clearing plaque) may not prevent further spread. On these bases, NDs are seen to operate as complex network failures that may not be addressed by **a** one-drug–one-target approach of pharmacotherapy.
- Heterogeneity in NDs is a common feature, with genotype and/or phenotype factors differentially affecting the presentation and severity of the disease, as well as making disease diagnosis and therapy difficult.
- The recurrent clinical failure in anti-amyloid therapies in AD with limited or inconsistent successes, as well as the limited symptomatic only relief in dopaminergic therapies in PD, underscore the limitation of a one-drug–one-target approach. Accordingly, we need to focus now a lot more on a paradigm shift toward multi-target strategies in NDs.

## 3. Pathophysiological Complexities of AD and PD

### 3.1. The Multi-Pathway Approach: AD Is Not a Single-Target Disorder

- The accumulation of senile plaque in AD

The accumulation of senile plaque and neurofibrillary tangle formation represent the pathological hallmarks of AD. Since the discovery of Aβ as the main component of the extracellular amyloid plaques back in 1984 [11], the amyloid hypothesis served as the leading theory of AD pathogenesis. Hence, drug discovery research aimed at reducing production and/or aggregation, or promoting disaggregation, took central stage for many decades. The homeostasis of Aβ formation from amyloid precursor protein (APP) through distinct cleavage pathways and enzymes (α-secretase, β-secretase, and γ-secretase) have been targeted by therapeutic agents. Several anti-Aβ monoclonal antibodies were also tested at the various stages of drug development trials. The frustration of adopting this approach was underpinned, however, by multiple failures of clinical trials that cast doubt on the clinical validity of the amyloid hypothesis of AD. The controversy of this therapeutic approach is further exacerbated by the demonstration of efficacy in preclinical assessments for these therapeutic agents. The heterogeneity of Aβ proteins in various forms, aggregates of various sizes (monomers, oligomers, and polymers), lack of direct correlation between Aβ deposits and cognitive impairment, and the limitation of animal/genetic models representing AD progression in human are among the suggested factors accounting to unpredictable outcomes. On the positive side, the recent evidence from anti-Aβ monoclonal antibody treatments showing a reduction in Aβ plaque coupled with delayed disease progression give promise for the Aβ-based therapy of AD. The key breakthrough herein is the FDA (The U.S. Food and Drug Administration) approval of lecanemab in 2023 [12] and donanemab in 2024 [13]. This also marks a shift in the AD therapy approach, moving from strictly symptomatic management to disease-modifying therapies.

- Tau protein hyperphosphorylation

Studies in the late 1980s [14,15] established that tau protein hyperphosphorylation is a primary pathological hallmark of AD, as intracellular tau aggregation leading to the formation of neurofibrillary tangles (NFTs) attributes to synaptic failure, neuronal dysfunction, and death. Several in vitro and animal studies explored the potential of phosphorylated-tau anti-aggregation approach as a therapeutic option for AD but with little clinical evidence of efficacy. Nonetheless, new drug development and repurposing based on tau hyperphosphorylation through inhibition of the key hyperphosphorylation enzyme, glycogen synthase kinase-3β (GSK-3β), by some agents such as tideglusib was tried out, though failed in late-stage clinical trials. Others which showed promise in preclinical studies but not in clinical trial include lithium (GSK-3β inhibitor), sodium selenate (protein phosphatase 2A activator to dephosphorylate tau), and saracatinib (tau phosphorylation inhibitor), among others. What is more important to drug discovery is that Aβ aggregation and accumulation can induce tau phosphorylation. For example, exposure of neuronal cell to Aβ causes an activation of p38 mitogen-activated protein kinase (p38) that leads to tau hyperphosphorylation both in vitro and in vivo [16]. This further gives insight that, as with the Aβ-based therapies, a single-target approach based on tau hyperphosphorylation may not lead to a breakthrough in the search for a cure for AD.

- Oxidative stress

A vast, compelling body of evidence overwhelmingly positions mitochondrial dysfunction at the very heart of both neuronal oxidative stress and the progression of NDs. For a comprehensive overview of this generally accepted background, readers are directed to review articles [17,18]. The increased level of mitochondria-based oxidative stress and dysfunction in ageing brain underpins the role of antioxidant-based therapies for AD. What is paramount to drug discovery is that oxidative stress and/or reactive oxygen species (ROS) promotes Aβ accumulation and tau hyperphosphorylation. On the other hand, Aβ aggregation generates more free radicals and induce neurotoxicity. While direct antioxidant therapies do not serve as silver bullets for AD therapy, targeting ROS production or boosting antioxidant defences in combination with other approaches will have benefits in AD therapeutics. Moreover, redox-active transition metals such as iron, copper, and zinc, which are known to be involved in AD pathology, induce oxidative stress directly as well as via interaction with Aβ. Hence, oxidative stress is not just a byproduct of AD but a driver of the pathologic process which initiate a feedback loop that increases both tau phosphorylation and amyloid plaque accumulation. As expected, however, the clinical efficacy of such a therapeutic target by its own is not yet validated as many antioxidants such as vitamins E and C have shown limited success in large human trials, again, despite success in animal studies. Hence, the future direction of research on antioxidants/mitochondria-based AD therapy should be based on a multi-targeted approach that combine mitochondrial/antioxidant support with anti-amyloid, anti-inflammatory drugs, and/or other anti-AD therapies (see below).

- Neuroinflammation

Along with oxidative stress, neuroinflammation is a central hallmark of NDs pathogenesis [17,19]. Microglial activation can be induced by a variety of agents in the brain including Aβ, resulting in the release of proinflammatory cytokines such as IL-1β, IL-6, and TNF-α, as well as ROS. There is growing evidence to suggest that pathogenic protein aggregation and neuroinflammation mutually promote neurodegeneration. Furthermore, activation of microglia by Aβ plaques in AD brains leading to the neuroinflammatory responses is known to be driven by the NLRP3 (nucleotide-binding domain, leucine-rich–containing family, pyrin domain–containing-3) inflammasome pathway in various experimental models [20]. The interlinking pathway between the various components of AD pathology is striking: Aβ accumulation induces oxidative stress, oxidative stress promotes tau pathology, and neuroinflammation exacerbates both.

- Synaptic dysfunction

Synaptic dysfunction is the strongest and among the most direct link to cognitive decline in AD. In fact, the overwhelming evidence in the scientific literature placed synaptic dysfunction as the primary, early, and invariant feature of AD, often appearing before significant neuron loss, amyloid plaque formation, or neurofibrillary tangle development. This takes a form of reduction in synaptic density and markers, Aβ and tau toxicity related to disrupting mitochondrial function and vesicle trafficking, plasticity deficits, and synaptic loss [21,22,23]. The above-mentioned pathologies, such as Aβ, tau pathology, neuroinflammation, mitochondrial impairment, and/or oxidative stress acting as self-reinforcing pathological network account to synaptic dysfunction. On this basis, targeting a single node (e.g., as discussed for amyloid hypothesis) in isolation appeared to be the reason for multiple failures of late-stage clinical trials over the years.

### 3.2. Parkinson’s Disease: Beyond Dopamine Deficiency

**Figure 1:**
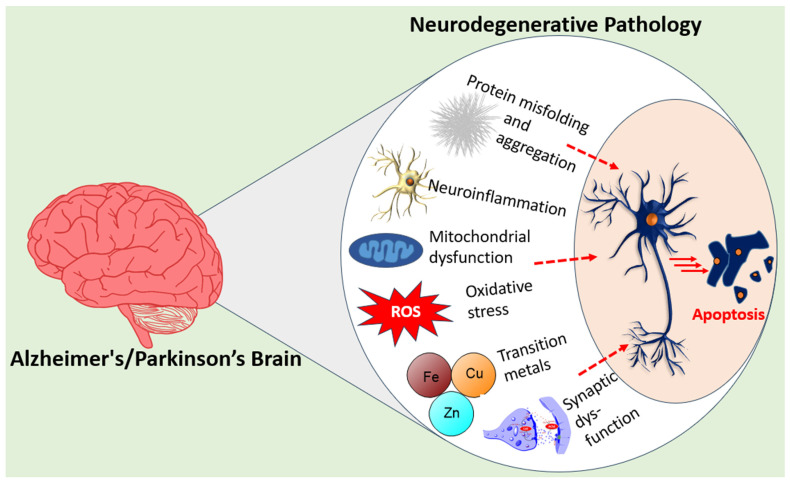
The complex pathological mechanisms involved in neurodegenerative diseases. Transition metals such as iron, copper, and zinc are key mediators of ROS generation under pathological conditions in the brain. The resulting oxidative stress coupled with mitochondrial dysfunction crosstalk with neuroinflammation orchestrated by activated glial cells in the brain. Protein aggregation such as α-synuclein in the form of Lewy bodies in PD or Aβ/tau in AD actively participate in oxidative stress, neuroinflammation, and mitochondrial dysfunction, leading to loss of synaptic plasticity and ultimately neuronal cell death. This interconnected, multifactorial network of pathological pathways, collectively contributing to NDs (as shown by arrows), makes a single-target approach to ND therapy ineffective. Aβ, amyloid beta; AD, Alzheimer’s disease; PD, Parkinson’s disease; NDs, neurodegenerative diseases; ROS, reactive oxygen species.

PD has traditionally been treated as a dopaminergic disorder owing to the deficit of this neurotransmitter resulting from the degeneration of dopaminergic neurons in the substantia nigra pars compacta. Clinical evidence suggests that by the time motor symptoms of PD such as rigidity, tremors, and bradykinesia appear, patients have typically lost at least 50 to 80% of these dopamine-producing cells in the substantia nigra [24]. The underlying mechanism of PD associated with neurodegeneration is often linked to the accumulation of protein clumps known as Lewy bodies, which consist of aggregated α-synuclein. Hence, there is a striking similarity of PD with AD as both pathologies are associated with disease-specific protein aggregation. Another characteristic feature of PD shared with AD are mitochondrial dysfunction and neuroinflammation. Hence, increased levels of ROS and oxidative stress in collaboration with microglial activation drive neuroinflammation via increased levels of proinflammatory cytokines (TNF-α, IL-1β, and IL-6) leading to neuronal cell death. In both PD and AD, impaired proteostasis as a convergent pathological mechanism is known to be evident as the failure of protein quality control systems (including the ubiquitin–proteasome system and autophagy–lysosome pathway) leads to the accumulation of misfolded proteins; α-synuclein in PD (Lewy bodies) and Aβ/tau in AD [25,26]. Hence, just like AD, PD is now recognised as a systems-level disease, requiring multi-target intervention. The overall pathological cascades of these NDs are depicted in Figure 1.

## 4. Natural Products: A Systems Pharmacology Solution to AD and PD

### 4.1. Chemical Diversity and Evolutionary Optimisation

Natural products (NPs) are specialised metabolites produced by living organisms (plants, mushrooms, microbes, marine life, etc.) that, through millions of years of evolution, have been refined to act as ligands for biological macromolecules. Their chemical diversity and structural complexity make them relevant pre-validated sources for drug discovery, with roughly 25–50% of approved drugs widely known to be NPs or their derivatives. The study by Newman and Cragg [27], for example, showed that about 32% of approved small molecules between 1981 and 2019 could trace their origin to NPs. These compounds are created to serve specific ecological functions such as defence against predators, competition with other organisms, or as signalling molecules in the regulation of metabolism which often translates into effective therapeutic agents such as antimicrobial, anticancer, antioxidant, and anti-inflammatory agents. It is thus fair to say that NPs, unlike synthetic compounds, are structurally optimised to interact with biological targets (e.g., proteins, enzymes, and receptors). When compared to synthetic compounds, NPs possess higher structural complexity, greater molecular rigidity and high proportion of sp^3^-hybridised carbons (stereo centres) in their molecules [28,29]. It has long been believed that this increased three-dimensional character is likely to improve pharmacodynamic and pharmacokinetic properties [30] and make NPs better suited for targeting complex molecular interactions, such as protein–protein interfaces. Hence, NPs occupy a different, often less explored, area of chemical space compared to synthetic combinatorial libraries.

Beyond their structural diversity and structural scaffolds as terpenoids, alkaloids, polyketides, flavonoids, etc., many NPs are known to interact with multiple proteins often exhibiting synergistic effects. Accordingly, NPs are inherently suited for polypharmacology, a key requirement for NDs treatment.

### 4.2. Multi-Target Mechanisms of NPs

As discussed in Section 5, many NPs even in their pure form are known to simultaneously act on multiple biological targets. This includes inhibition of oxidative stress pathways, inflammatory signalling, protein aggregation, and mitochondrial dysfunction. An interesting development in recent years is evidence showing many NPs increasing the level of brain-derived neurotrophic factor (BDNF) coupled with promoting neurogenesis and synaptic plasticity. Hence, through the therapeutic principle of polypharmacology, NPs may not only suppress the pathological sequel of neurodegeneration but could also potentially restore normal physiology by replacing dead neurones through neurogenesis. Interestingly, many NPs known to show benefit through multiple effects in NDs such as curcumin have been shown to upregulate BDNF expression while promoting hippocampal neurogenesis and synaptic plasticity [31].

### 4.3. Network Pharmacology Evidence

A recent (2026) network pharmacology study (based on computational studies constructed a network of 17,869 human proteins and 1496 FDA-approved drugs to assess the potential of NPs as drugs [32]. The study showed that dietary flavonoids target on the average 45.3 proteins per compound, compared to just 16.8 for conventional FDA-approved drugs. Based on this study, it was concluded that flavonoids have the potential to interact with ~2–3-fold more targets than conventional drugs. This finding directs our attention to the general trend of many NPs modulating the entire biological networks rather than single proteins (one-drug–one-target approach). The study by Liang et al. [33] employing pathway-based network medicine constructed an AD-specific gene–pathway network and identified purified NPs, (−)-vestitol and salviolone, as key multi-target candidates. Subsequent in vivo validation in *APP/PS1* mice demonstrated synergistic cognitive improvement and reduction in Aβ pathology through modulation of Ca^2+^ signalling and neuroactive ligand–receptor pathways [33]. Another network pharmacology-based investigation was that by Zhang et al. [34] which identified the isoquinoline alkaloid, berberine, acting on core targets including APP and proliferator-activated receptor gamma (PPAR-γ), with experimental data confirming regulation of amyloid processing and neuroinflammatory signalling pathways. By combining network pharmacology with molecular docking and subsequent validation via in vitro study, the active constituents of *Rosmarinus officinalis* were identified (e.g., carnosic acid and rosmarinic acid) to target apoptosis- and inflammation-related pathways in AD [35].

Since hundreds of proteins may be simultaneously targeted, the use of NPs for AD or PD supports their role as systems-level therapeutics. For NPs like flavonoids, their synergistic effect in various bioactivity studies is also evidently shown in network-predicted association with disease conditions. Several studies employing network pharmacology in crude traditional herbal medicines also assisted in the identification of active ingredients acting through multi-target, multi-pathway mechanisms in PD [36,37,38,39] or AD [40,41,42,43] treatment.

## 5. Emerging Natural Product-Based Strategies over the Last 6 Years

In 2019, my review article [43] on the promise of natural products for NDs therapy was published in *Molecules* under the heading: “Natural Products in Alzheimer’s Disease Therapy: Would Od Therapeutic Approaches Fix the Broken Promise of Modern Medicines?”. The article highlighted the potential of phytochemicals to target multiple pathological pathways in AD such as Aβ accumulation, tau hyperphosphorylation, oxidative stress, and neuroinflammation as a promising, often safer alternative to synthetic drugs. Key examples of natural products acting through polypharmacology principles presented in the article included resveratrol, curcumin, caffeic acid phenethyl ester, capsaicin, berberine, nordihydroguaiaretic acid, quercetin, myricetin, morin, catechin, hyperforin, ginsenoside compound k, epicatechin-3-gallate, astaxanthin, and docosahexaenoic acid. Since then, we have seen a shift from traditional phytochemicals to engineered, clinically relevant candidates. The main focus of this progress can be summarised as follow:

- Focus on bioavailability: Most research is now focusing on nanoparticle-based carriers to increase stability and specificity of NPs.
- Targeting neuroinflammation: NPs like curcumin are now being used to specifically target the NLRP3 inflammasome, a key driver of AD and PD progression.
- Focus on emerging new technologies: Systems biology integrating multi-omics data (genomics and proteomics) are used to identify novel therapeutics, while artificial intelligence (AI) such as machine learning and deep learning are employed for virtual screening of large NPs libraries, predicting drug–target interactions, and optimisation.
- Combination therapies: Research is shifting towards combining NPs with conventional medicine (e.g., memantine) or other NPs to enhance efficacy.

A brief highlight of this progress is presented below with some exemplary NPs.

### 5.1. Epigallocatechin Gallate (EGCG)

**Figure 2:**
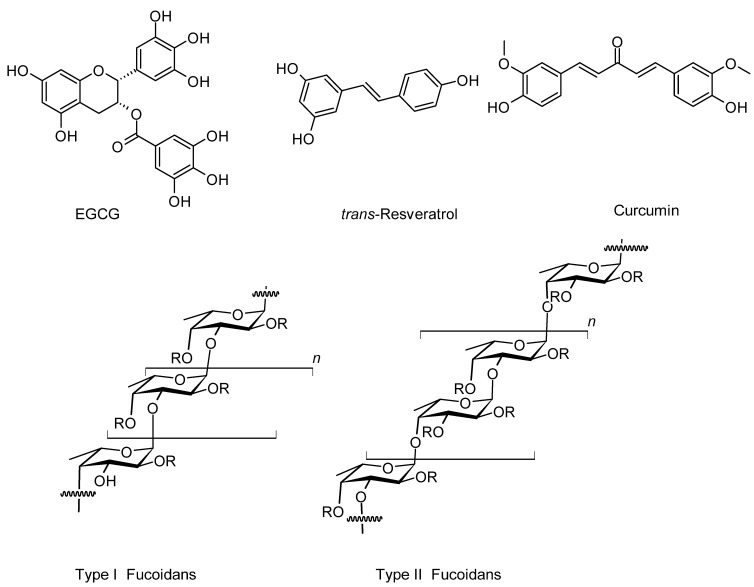
Structures of EGCG, *trans*-resveratrol, curcumin, and fucoidans. The three polyphenols continue to dominate research on NPs as potential therapies for neurodegenerative diseases such as AD and PD. The structures of fucoidans with two known backbones are shown with R groups representing fucopyranose, glucuronic acid, and sulphate groups. Note that galactose, mannose, xylose, rhamnose, arabinose, and glucose units do also occur in seaweeds in various forms with their location still not established. The type (I) fucoidans possess repeated (1→3)-l-fucopyranose backbone, while type (II) fucoidans possess alternating and repeated (1→3)- and (1→4)-l-fucopyranose backbone.

Epigallocatechin-3-gallate (EGCG, Figure 2), the primary bioactive polyphenol in green tea, has emerged as a promising therapeutic agent for NDs due to its extensive neuroprotective properties and ability to modulate neuronal survival through a polypharmacology principle. Along with resveratrol and curcumin (see below), EGCG represent a transformation of supplements to drug platform in NDs. As outlined in my preceding review [43], rather than targeting a single pathological mechanism, EGCG exerts its beneficial effects by simultaneously reducing oxidative stress, protecting mitochondrial function, and modulating key signalling pathways involved in cell survival and death. Through a potent antioxidant effect, EGCG neutralises ROS and reduces oxidative damage in neuronal tissues. Concurrently, it is widely known to stabilise mitochondrial membrane potential, thereby preventing the release of pro-apoptotic factors, while also supports mitochondrial biogenesis in neuronal tissues. Furthermore, EGCG acts on multiple signalling cascades including the activation of pro-survival pathways (e.g., PI3K/Akt) while inhibiting pro-apoptotic processes through polypharmacological capacity to combat the complex diseases like AD and PD. On the clinical trial field, a 10-month intensive multimodal lifestyle population-based intervention study was conducted in Spain with 129 participants (APOE-ε4 carriers aged 60–80) with subjective cognitive decline receiving a green tea extract enriched with EGCG [44]. A significant cognitive benefit was observed during the study period as well as the 3 months washout period.

Despite this therapeutic potential, the clinical application of EGCG has been constrained by poor metabolic stability, low bioavailability, and limited blood–brain barrier (BBB) permeability. Consequently, recent studies have shifted focus towards advanced nanotechnology to enhance EGCG delivery to the central nervous system [45,46,47]. Such studies now heavily focus on developing nanoformulations, such as polymeric nanoparticles (e.g., PLGA), nanoliposomes, and lipid-based carriers, which improve EGCG’s pharmacokinetic characteristics and protect it from rapid degradation. These nanotechnology-based strategies, including surface modification with targeting peptides, have demonstrated superior bioavailability, improved BBB transport, and higher therapeutic efficacy in NDs models when compared to free/unformulated EGCG [48]. This transformation reflects a broader trend of re-engineering NPs such as food supplements into drug platforms.

### 5.2. Resveratrol

Another multi-target or polypharmacological natural compound is resveratrol (Figure 2) with neuroprotective effects associated with general antioxidant effect, amelioration of mitochondrial dysfunction, and anti-inflammatory effects with proven mechanism of suppressing microglial activation and NF-κB–mediated cytokine production [49]. As with many polyphenolic compounds showing a promise for AD and PD, resveratrol also modulates Aβ pathology by inhibiting aggregation and enhancing clearance of both Aβ and α-synuclein [50,51,52]. Interestingly, combination of resveratrol and curcumin with anti-amyloid monoclonal antibodies (aducanumab and lecanemab) has been shown to lead to a greater degree of inhibition of Aβ aggregation [53].

Despite the numerous in vitro and in vivo evidence demonstrating the potential of resveratrol in AD and PD therapies, clinical trial-based research on this compound remains very scarce. Many studies are still citing the old data from the 2015 study of phase 2 trial on individuals with mild-moderate AD which outlines that it is safe and well tolerated [54]. A randomised, double-blind trial on *trans*-resveratrol in mild-to-moderate AD was conducted on a small group of people (15 participants per group) receiving treatment for 52 weeks [55]. A small margin of benefit based on reduction on neuroinflammation markers were observed. A systematic review and meta-analysis by Jin et al. [56] shows that clinical efficacy is generally inconsistent with no strong evidence of cognitive improvement by resveratrol. Hence, the available clinical data is still insufficient to make a conclusion on the therapeutic merit of resveratrol for AD. There is also no high-quality data on the clinical trial of resveratrol for PD to cite in the last 6 years. The low bioavailability of resveratrol along with its rapid alteration by phase I and II enzymes across multiple tissues are often cited [57] as major limiting factors for its potential as a therapeutic agent. In fact, a recent human pharmacokinetic meta-analysis data by Szymkowiak et al. [58] showed that, despite efficient absorption, systemic availability of free resveratrol remains extremely low (mean peak serum concentration (C_max_) ≈ 31 ng/mL) due to rapid phase II metabolism into glucuronide and sulphate conjugates. Interestingly, a randomised crossover study [59] of novel solid formulations in healthy Chinese subjects reported significant increases in absorption rate, extent, and relative bioavailability (C_max_ and area under the curve) of resveratrol. This confirms that formulation strategies can partially overcome poor bioavailability but the overall bioavailability to the brain remains suboptimal. Other advances on resveratrol formulation based on solid dispersion technologies showed enhanced solubility and oral absorption [60]. Overall, these findings establish that while resveratrol has strong neuroprotective mechanisms relevant to AD and PD, its clinical translation is constrained primarily by pharmacokinetic barriers, and hence recent progress has shifted toward delivery optimisation as the critical determinant of therapeutic efficacy for NDs.

### 5.3. Curcumin

Curcumin (Figure 2), a polyphenolic compound primarily isolated from turmeric, has long known to possess significant potential as a therapeutic agent for both AD and PD due to its multi-target neuroprotective properties. This is based on the established role of curcumin mechanistically acting as antioxidant and anti-inflammatory effects, modulating neuroinflammation, and interfering with pathological protein aggregation. The recent exemplary study using Aβ-induced AD model in mice revealed that the effect of curcumin in improving memory deficit is linked to amelioration of neuroinflammation and oxidative stress [61]. With relevance to PD, experimental models such as 6-hydroxydopamine rat model were used to establish the dosage for improvement of movement symptoms observed in the studies [62]. The anti-aggregation properties of curcumin such as that against Aβ was also well established, and can be further enhanced through chemical modifications [63]. It is worth noting that these properties of curcumin through crosstalk between antioxidant properties and inhibition of Aβ aggregation is shared by resveratrol [64], suggesting a common polypharmacology pathway of mechanism of action for these phenolic compounds.

As with many NPs, the clinical translation of curcumin is significantly limited by its poor bioavailability. The in vivo AD study by Phuna et al. [65] using poly lactic-co-glycolic acid (PLGA) nanoparticle formulations specifically showed that the nanoformulation significantly improved pharmacokinetics and cognitive outcomes when compared to free curcumin. Similarly, the study by Cai et al. [66] in vitro and rotenone-induced PD mouse model explored the mechanism of action of curcumin prepared in advanced formulations (e.g., solid lipid curcumin particles). They have shown that such formulations can improve bioavailability up to 100-fold. The randomised controlled trial by Das et al. [67] highlighted that specialised formulations (CurQfen^®^) were required to enhance absorption and BBB penetration, hence confirming that unmodified curcumin has insufficient bioavailability for meaningful neurological effects. A single-centre crossover study using twelve healthy adults showed that a 178-fold bioavailability increase over standard curcumin can be achieved via solvent-free co-grinding preparation [68]. A study by Gupta et al. [69] using curcumin encapsulated lipidic nanoconstructs further revealed a 69.78 times higher oral bioavailability in rats when compared to unformulated free curcumin. Thus, all the available data indicate that, although curcumin can modulate key pathological pathways in AD and PD, its poor solubility, rapid metabolism, and low brain bioavailability remain major barriers, necessitating advanced formulations to achieve clinically relevant efficacy.

### 5.4. Fucoidan from Marine Macroalgae

Fucoidan is a sulphated polysaccharide isolated from brown algae and sea cucumbers. It is widely used in nutritional supplements and cosmetic products for its claimed antioxidant, anti-inflammatory, and antitumor and neuroprotective properties, among others. Structurally, these group of polysaccharides are rich in fucose and constructed with two types of backbones: type (I) with repeated (1→3)-l-fucopyranose, and type (II) with alternating and repeated (1→3)- and (1→4)-l-fucopyranose. The type II fucoidan isolated from *Fucus vesiculosus* has been shown to display neuroprotective effects in an 1-methyl-4-phenyl-1,2,3,6-tetrahydropyridine (MPTP)-induced PD mouse model [70]. Based on this animal model, it was reported to reverse mitochondrial dysfunction, inhibit neuronal apoptosis, and reduce dopaminergic neuron loss in vivo. This data is constant with the effect of this compound in neuroinflammation and neuroprotection in other animal models of diseases such as colitis-neuroinflammation and anxiety [71] and scopolamine-induced AD rat [72] models. These studies highlight the growing trend of research on marine NPs including polysaccharides as potential therapies for NDs. Clinical evidence validating these periclinal promises however remains to be seen.

### 5.5. Clinical Evidence: Crude Plant Extracts

Advances in NPs research for AD and PD through mechanistic understanding of multi-target activity continued to include crude drug preparations from various sources. A randomised, double-blind parallel-group clinical trial in 2026 [73] assessed the efficacy of hydroalcoholic extract of *Myrtus communis* L. in patients with mild-to-moderate AD. Only fifty patients were used in the four-week trial study but the results revealed a statistically significant improvement in cognitive function. Hence, beyond the numerous in vitro and in vivo effects of relevance to neuroprotection, the result validated previous findings on animal models of AD for the crude plant extract [74,75] or its active ingredient, myrtenal [76]. This kind of research providing clinical validation to NPs with preclinical promise is a necessary step to bring a breakthrough in this field. It remains the case, however, that outcomes in patients are heterogeneous across study designs and populations. Other examples include *Ginkgo biloba* extract (EGb 761) demonstrating a statistically significant improvements in cognition and neuropsychiatric symptoms in patients with mild-to-moderate AD [77,78]. *Mucuna pruriens* is a natural source of levodopa, which was reported to improve rapid motor symptom of PD and distinct pharmacokinetic advantages compared with conventional formulations [79].

## 6. Translational Success: From Natural Products to Drugs

### 6.1. Lessons from the Phenserine Lineage

**Figure 3:**
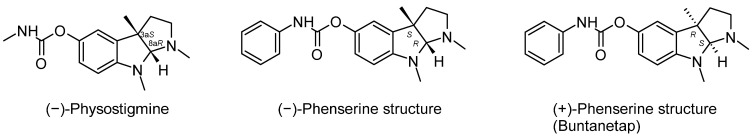
Structures of physostigmine, phenserine, and buntanetap. Notice the additional benzine ring in phenserine vs. physostigmine and the stereochemistry difference between phenserine and buntanetap as mirror images.

The evolution of drug discovery from physostigmine to buntanetap through phenserine (physostigmine → phenserine → buntanetap, Figure 3) is a good example of transformation from single-target AChE inhibition to multi-target protein regulation. This trajectory exemplifies how NPs serve as drug discovery scaffolds:

Physostigmine as a naturally occurring alkaloid in its minus isomer form (3a*S*, 8a*S* positions, Figure 3) is derived from Calabar beans and acts as a short-acting, reversible AChE inhibitor. While it increases ACh levels to aid memory, its clinical use for AD was limited by a very short half-life, low bioavailability, and significant gastrointestinal side effects (e.g., nausea and vomiting).

Phenserine ((−)-phenserine) is a derivative of physostigmine that also acts as a potent, long-acting inhibitor of AChE. It was designed to improve the physostigmine’s stability and duration of action. Crucially, phenserine was able to reduce the translation of Aβ precursor protein, APP, offering a secondary, disease-modifying mechanism beyond just symptomatic cholinergic action [80]. It was one of those anti-AD therapy, however, which failed in late-stage clinical trial in the early 2000s.

Buntanetap is the pure (+)-enantiomer of phenserine (Figure 3). While phenserine is the active inhibitor of AChE, buntanetap has weak AChE activity but is highly effective at reducing the translation of APP. Buntanetap is currently being investigated for the treatment of AD and PD due to its ability to lower multiple neurotoxic proteins (Aβ, tau, α-synuclein, and TDP43). In vitro evidence showing these effects [81] as well as in vivo experiments using transgenic mouse models [81,82] have been published. Hence, buntanetap represents a new generation of drugs derived from NPs scaffolds. A double-blind, placebo-controlled, multi-centre study in the US showed that it was safe and well tolerated when administered in AD and PD patients at 80 mg [83]. In 2025, Annovis announced that its phase 2/3 AD study on buntanetap revealed key targets of neuroinflammation and neurodegeneration cascades and classed it as potential disease-modifying agent beyond symptomatic relief [84]. The compound is known to act as follows, suggesting a systems-level mechanism, rather than single-target inhibition:

- Inhibits translation (by blocking translation of mRNA) of multiple neurotoxic proteins. For example, it binds to the iron-responsive element of APP mRNA.
- Targets AChE, Aβ, tau, α-synuclein, and others simultaneously.
- Currently in Phase III trials for AD [85]. The safety profile of the drug including that based on clinical trial is now published [83].

### 6.2. Other Examples of Translational Success

**Figure 4:**
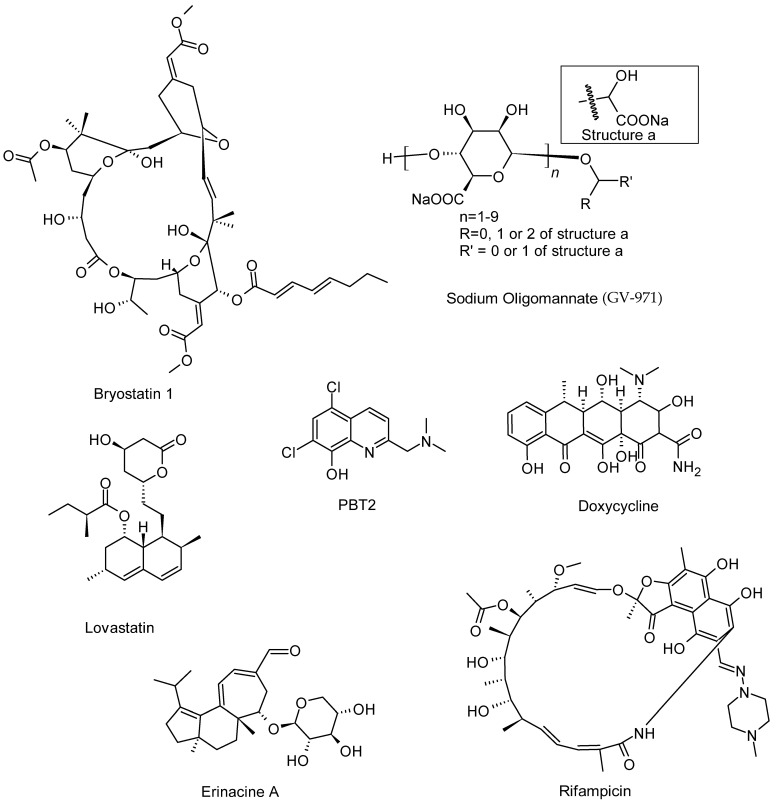
Structures of some NPs that show effects under clinical trials for AD and/or PD.

Another clinical study worth mentioning is sodium oligomannate (GV-971, Figure 4), a marine-derived oligosaccharide (from brown algae), which was shown to modulate neuroinflammation via the gut–brain axis, while stabilising cognitive decline in AD patients [86]. Bryostatin-1 (Figure 4) is a macrocyclic lactone isolated from marine bryozoans which has progressed into clinical investigation. The primary trial data demonstrated mechanism of action via activation of protein kinase C signalled a marginal improvement in synaptic function and cognitive outcomes in AD subjects [87]. At preclinical studies, many other evidence of efficacy for many NPs such as fucoxanthin [88], gracilin A [89], and dieckol [90], is emerging but their clinical efficacy is yet to be demonstrated. Lovastatin (Figure 4) is a cholesterol-lowering agent originally derived from the fungus *Aspergillus terreus*, which has been evaluated in observational and interventional clinical studies for its potential neuroprotective effects in AD and PD. The data suggest the associations between statin use (the most potent protective agent being rosuvastatin) and reduced risk of cognitive decline, along with reduction in neuroinflammation and improved cerebral vascular function [91]. Another promising fungal-derived compound is cerebrolysin, a peptide derived from enzymatic breakdown of porcine brain proteins with fungal fermentation. In a study using AD patients on its own or in combination with conventional drugs (e.g., donepezil), it demonstrated modest improvements in cognitive outcomes [92], but more research is needed to validate its clinical potential. Of the microbial-derived NPs reaching clinical trial for AD and/or PD in the last six years include PBT2 (5,7-dichloro-2-[(dimethylamino)methyl]-8-hydroxyquinoline, Figure 4), a synthetic 8-hydroxyquinoline compound derived from microbial metal-chelating scaffolds. Its phase II clinical trials on AD back in 2010 showed promise, though widely considered as unsuccessful [93], and since then not much data has come through apart from mechanistic studies such as its interaction with Aβ [94]. The tetracyclines antibiotic doxycycline (Figure 4) for inhibition of α-synuclein aggregation and reducing inflammatory markers in preclinical studies has attracted interest, but so far limited benefits have been reported under clinical trial, such as reduction of dyskinesia in PD patients [95]. Rifampicin (Figure 4) has a similar profile in preclinical studies, where it was shown to suppress Aβ oligomer accumulation and tau hyperphosphorylation, along with improvement in memory performance in animal models (e.g., [96,97]). However, not much progress has been recorded through clinical trial studies. A classic example of NPs isolated from mushroom was erinacine A (Figure 4), a diterpenoid isolated from *Hericium erinaceus*, which showed a positive outcome under pilot clinical trial study employing erinacine A-enriched mycelium extract [98]. Another pilot study using a small number of participants was conducted by Bizjak et al. [99] where supplementation of diet with erinacine A-enriched mycelium extract showed improvements in cognitive function and changes in circulating BDNF level. Mushroom-derived β-glucans and polysaccharide fractions from various plant sources are being investigated under various experimental models of NDs. For example, supplementation of β-glucans from plants and yeast sources in experimental animals have shown a reduction in inflammatory status in the CNS (including the hippocampus,) and memory improvement via modulation of the gut microbiota [100,101]. The clinical translation of these NPs remains to be seen.

Curcumin and its formulated or derivatised analogues have entered clinical studies in AD patients although the results so far revealed with modest clinical efficacy with further caution of limited and rather inconsistent data. The pre-2019 research in this field has been summarised in the meta-analysis study by Voulgaropoulou et al. [102] with nothing substantial added since then. The same goes for resveratrol, while EGCG research has advanced into registered and completed clinical trials in related NDs such as multiple system atrophy [103]. As already described, a population-based study conducted in Spain showed that the supplement showed 2.6 times increase in cognitive improvement [44]. Given the numerous in vitro and animal studies on ginsenoside derivatives (e.g., Rg1) as standardised or chemically enriched formulations reported improvements in cognitive function and dopaminergic activity, there is call to move this research into clinical trial.

## 7. Enabling Technologies Driving Drug Development

### 7.1. Nanotechnology and Drug Delivery

As outlined in Section 5 for the key NPs with promising potential for NDs, their limitation is associated with their poor bioavailability. As with many conventional therapies, their limited ability to cross the BBB is also challenging. Compounds like curcumin, EGCG, and resveratrol suffer from low aqueous solubility and instability in physiological conditions, leading to rapid metabolism and clearance. For example, curcumin undergoes extensive hepatic and intestinal first-pass metabolism leading to glucuronate and sulphate metabolites [104], EGCG is chemically unstable and rapidly oxidised [105], and resveratrol is quickly metabolised into conjugated forms [106], all of which significantly reduce systemic exposure. Their BBB penetration is further constrained by their physicochemical properties, moderate to low lipophilicity, and molecular size. Another common problem for many drugs is active efflux by transporters such as P-glycoprotein, which limits drug availability in the brain and remains a major barrier to treating NDs.

Encapsulating NPs in nanoparticles to enhance their stability, solubility, and brain-specific delivery have been taking central stage in bioavailability studies. Readers can direct themselves to progress for each group of compounds formulated as polymeric nanoparticles (e.g., PLGA, and chitosan), lipid-based nanoparticles (liposomes, SLNs, and NLCs), metallic nanoparticles (e.g., selenium or gold), dendrimers, nanogels, etc. These technologies will undoubtedly drive future research on NPs for NDs. On these bases, successful clinical translation of NPs for NDs depends on addressing the following factors:

- Optimising pharmacokinetics through formulation strategies (e.g., nanoparticles, liposomes, or prodrugs) can enhance stability, absorption, and delivery to the brain.
- The restrictive architecture of the BBB permits primarily passive diffusion of lipid-soluble compounds with molecular weights typically below 400–600 Da [107]. Therefore, improving BBB permeability requires careful optimisation of lipophilicity and molecular size, alongside strategies that exploit carrier-mediated transport systems to facilitate brain uptake.
- Integrating these natural compounds into advanced platforms such as hybrid molecules may enhance potency and specificity at lower doses.
- Advances in biomarker-driven validation along with pharmacokinetic profiling may confirm the level of NPs target engagement in the brain.
- Standardisation of crude NPs and scalable synthesis are essential to ensure reproducibility and regulatory approval.

Overcoming these interconnected challenges is critical for translating promising NPs into effective therapies for NDs.

### 7.2. Systems Biology and AI

Systems biology and AI are on the verge transforming NDs research from a focus on single-target treatments to a holistic understanding of the disease. These technologies will enable precision medicine and earlier diagnosis of complex NDs. The capability of AI allows rapid analyses of vast, heterogeneous, and multi-scale datasets in areas of genomics, proteomics, transcriptomics, metabolomics, and neuroimaging. This creates ideal environment to identify intricate patterns (e.g., relationships, anomalies, and correlations), while systems biology builds models of gene regulation networks to reveal how these molecular changes drive NDs. 

Emerging research increasingly integrates AI-guided design to optimise efficacy and target specificity of NPs such as flavonoids. Some in vitro and in silico studies have already demonstrated that specific flavonoids can directly inhibit α-synuclein fibrillation by targeting protein aggregation at the molecular level [108,109]. The study by Noori et al. [110] further validated the potential of advanced AI-driven frameworks in drug discovery where a graph-based AI system in NDs can generate drug candidates. Hence, AI-guided design will enhance the discovery and optimisation of NPs, offering a promising and increasingly translational strategy for developing novel treatments for AD and PD.

Kim et al. [111] employed an integrated in silico virtual screening workflow combining small molecular weight NPs with molecular docking, molecular dynamics, and pharmacokinetic predictions. Through this approach, they identified compounds such as baicalein and genkwanin which target Aβ and tau aggregation. With relevance to PD, Horne et al. [112] demonstrated that machine learning-guided molecular design can identify small molecules capable of inhibiting α-synuclein aggregation.

Mottaqi et al. [113] outlined an approach to integrate AI with multiomics systems biology and electronic health record data mining for personalised drug repurposing in AD. On this basis, it was possible to elucidate the molecular diversity of AD and predict promising drug repurposing opportunities. This was based on harmonised transcriptomic (bulk RNA-seq) and genomic (genome-wide association study) data from 2105 brain samples sourced from three independent studies. Hence, modern drug discovery on NPs integrating network pharmacology and machine learning will allow the identification of multi-target compounds and prediction of synergistic effects which are common in NPs pharmacology. Readers are also directed to a timely review article on AI-driven approach in AD research [114,115]. For practical application of AI in NPs-based drug discovery and development, readers are directed to a review article by Othmana et al. [116].

### 7.3. Combination Therapies

Another approach reflecting a shift toward network modulation rather than single-node inhibition in drug discovery for NDs is a combination therapeutic approach. Natural products are increasingly used in multi-compound formulations with a principle of synergistic drug combinations. Tracing back to the use of NPs in traditional medicine, such as traditional Chinese medicine (TCM) or Ayurveda, a mix of several plant species are used in their formulations. In addition to such natural plus natural combinations, the combination of NPs with conventional drugs is possible.

Gomez-Sequeda et al. [117] showed that a combination of tramiprosate, curcumin, and a kinase inhibitor (SP600125) significantly reduced Aβ accumulation and improved neuronal phenotype in PSEN1 mutant cholinergic-like neurons. Similarly, Xie et al. [118] demonstrated in a triple-transgenic AD mouse model where co-administration of curcumin with a synthetic analogue of coenzyme Q (mitoquinol mesylate, MitoQ) improved cognitive performance and attenuated neuropathology, including oxidative stress and mitochondrial dysfunction, when compared to single treatments. The rationale for using MitoQ in this experiment was based on its positive charge, which allows it to selectively accumulate in negatively charged mitochondria, making it several hundred times more efficient than coenzyme Q. The in vivo study by Xie et al. [118] demonstrated that co-administration of a mitochondrial-targeted antioxidant (MitoQ) with curcumin improved cognitive performance and reduced neuroinflammation and mitochondrial dysfunction in AD models, further supporting the concept that NPs can potentiate conventional or clinically developed therapeutic strategies. Combination studies based on withanone, apigenin, bacoside A, baicalin, and thymoquinone indicated that the compounds displayed a greater degree of potency than the monotherapies, leading to a significant reduction in age-related motility defects *Caenorhabditis elegans* AD model [119]. In an Aβ-model of AD in rats, co-administration of rivastigmine and artesunate resulted in synergistic cognitive and neuroprotective benefits along with improvement in oxidative stress and inflammatory markers [120].

## 8. Challenges and Future Directions

As a summary to the advantages of NPs in NDs, they allow simultaneous modulation of multiple pathways (multi-target efficacy), they are evolutionarily optimised for biological systems (reduced toxicity), they are unique scaffolds not found in synthetic libraries (structural diversity), and they are aligned with multifactorial disease models (compatibility with systems medicine). Despite this promise, however, several challenges remain in pharmacokinetics areas (poor solubility and rapid metabolism), standardisation issues in variability in plant extracts, limited large-scale trials (clinical translation), and regulatory complexities including in classification of drug vs. supplements.

New technologies are now positioned to transform future drug discovery approaches for NDs. For example, PROTAC (proteolysis-targeting chimera) technology enables the selective degradation of pathogenic proteins rather than conventional inhibition, thereby addressing protein aggregation more effectively. By integrating structurally diverse NPs into PROTAC design, potent degraders with improved selectivity and efficacy, may be identified [121]. Furthermore, advances in proteomic techniques enable systematic identification and validation of protein targets and degradation pathways, facilitating the discovery of novel mechanisms of action and optimising PROTAC constructs derived from NPs [122]. These innovations create a powerful platform that bridges NPs chemistry and targeted protein degradation to accelerate the development of new therapeutics for NDs.

Based on the recent trends, hybrid molecules combining natural scaffolds and synthetic modifications, precision medicine which allows tailoring treatments based on patient-specific pathology, network-based drug design targeting entire disease networks, and integration with biologics to combine NPs with antibodies or gene therapies will offer opportunities for the future drug discovery for NDs.

## 9. Conclusions

The last decade, and especially the past six years, has marked a critical transition in drug discovery for NDs. The failure of the single-target paradigm underscored the need for therapies that address the complex, interconnected nature of diseases like AD and PD. Natural products offer a unique and powerful solution to the existing problem as their inherent polypharmacology, combined with advances in nanotechnology, AI, systems biology, and medicinal chemistry, positions them as a central pipeline for next-generation therapeutics. Emerging examples such as engineered flavonoids, and especially buntanetap illustrate a new paradigm, from symptom management to disease modification, from single targets to network regulation. In this evolving landscape, NPs are no longer viewed as merely historical curiosities or alternative remedies, but rather becoming the foundation of a new era in NDs drug discovery.

## Data Availability

No new data were created or analyzed in this study. Data sharing is not applicable to this article.
